# Mapping healthcare utilization of people with Alzheimer’s Disease and Related Dementias to identify priority areas in the State of South Carolina

**DOI:** 10.1002/alz.093244

**Published:** 2025-01-09

**Authors:** Sara Knox, Mary Dooley, Jada Johnson, Kit Simpson

**Affiliations:** ^1^ Medical University of South Carolina, Charleston, SC USA

## Abstract

**Background:**

Alzheimer’s Disease and related dementias (ADRD) are a critical healthcare crisis in the State of South Carolina (SC), with over 115,000 individuals diagnosed with ADRD accounting for 11% of South Carolinians aged 65 or over and 52% of South Carolinians aged 85 or over. Exorbitant resources are used to care for these individuals, including $650 million in Medicaid dollars and over 300 million hours of unpaid caregiver time. SC has enacted a statewide plan to address ADRD with the mission of promoting “a comprehensive approach to risk reduction, early detection and diagnosis, high‐quality dementia services, and a coordinated and equitable continuum of care across…” Yet, ADRD does not present uniformly across SC. Heat mapping healthcare utilization of individuals with ADRD across SC provides an opportunity to better understand where resources should be directed to support individuals and caregivers currently coping with ADRD as well as inform future research aimed at risk reduction and disease prevention.

**Method:**

We used the South Carolina All‐Payer (Hospital and Emergency Department Admissions) Encounter data from the South Carolina Revenue and Fiscal Affairs Office to map the occurrence of Alzheimer’s disease and related dementias at the county level between 2018‐2020. Counties were grouped into five recognized regions of SC, 1) Upstate, 2) Midlands, 3) Coastal, 4) Pee Dee, and 5) Low Country. The Chronic Conditions Warehouse algorithm for ADRD was used to identify ICD‐10 codes for ADRD. Rates per 10,000 were reported.

**Result:**

The prevalence of ADRD was concentrated in two regions of the state, the Pee Dee region and the Upstate region. The Pee Dee region had the highest rates, with 9 of it’s 11 counties having rates of greater than 500/10,000. The eleven counties in the Upstate region had rates greater than 400/10,000. In the other three regions, counties typically had rates between 170‐ 300/10,000.

**Conclusion:**

The findings from this study provide clear evidence that there is a greater need for resources to support SC residents with ADRD and their caregivers in the Pee Dee and Upstate regions. Future studies should examine environmental, sociodemographic, and economic factors of these regions that may contribute to increased disease prevalence.

